# Topical Application of Double-Stranded RNA Targeting 2b and CP Genes of *Cucumber mosaic virus* Protects Plants against Local and Systemic Viral Infection

**DOI:** 10.3390/plants10050963

**Published:** 2021-05-12

**Authors:** Maria C. Holeva, Athanasios Sklavounos, Rajendran Rajeswaran, Mikhail M. Pooggin, Andreas E. Voloudakis

**Affiliations:** 1 Laboratory of Bacteriology, Scientific Directorate of Phytopathology, Benaki Phytopathological Institute, 14561 Kifissia, Greece; m.holeva@bpi.gr; 2 Laboratory of Plant Breeding and Biometry, Department of Crop Science, Agricultural University of Athens, 11855 Athens, Greece; thansclav@yahoo.gr; 3 Office of Rural Development and Inspections of Kephalonia, Ministry of Rural Development and Food, 28100 Argostoli, Greece; 4 Department of Biology, Swiss Federal Institute of Technology (ETH), Universitätsstrasse 2, 8092 Zürich, Switzerland; r.rajeswar@gmail.com; 5 PHIM Plant Health Institute, University of Montpellier, 34980 Montpellier, France; mikhail.pooggin@inrae.fr

**Keywords:** *Cucumber mosaic virus*, RNAi, double-stranded RNA, dsRNA vaccination, small interfering RNAs

## Abstract

*Cucumber mosaic virus* (CMV) is a destructive plant virus with worldwide distribution and the broadest host range of any known plant virus, as well as a model plant virus for understanding plant–virus interactions. Since the discovery of RNA interference (RNAi) as a major antiviral defense, RNAi-based technologies have been developed for plant protection against viral diseases. In plants and animals, a key trigger of RNAi is double-stranded RNA (dsRNA) processed by Dicer and Dicer-like (DCL) family proteins in small interfering RNAs (siRNAs). In the present study, dsRNAs for coat protein (CP) and 2b genes of CMV were produced in vitro and in vivo and applied onto tobacco plants representing a systemic solanaceous host as well as on a local host plant *Chenopodium quinoa*. Both dsRNA treatments protected plants from local and systemic infection with CMV, but not against infection with unrelated viruses, confirming sequence specificity of antiviral RNAi. Antiviral RNAi was effective when dsRNAs were applied simultaneously with or four days prior to CMV inoculation, but not four days post inoculation. In vivo-produced dsRNAs were more effective than the in vitro-produced; in treatments with in vivo dsRNAs, dsRNA-CP was more effective than dsRNA-2b, while the effects were opposite with in vitro dsRNAs. Illumina sequencing of small RNAs from in vivo dsRNA-CP treated and non-treated tobacco plants revealed that interference with CMV infection in systemic leaves coincides with strongly reduced accumulation of virus-derived 21- and 22-nucleotide (nt) siRNAs, likely generated by tobacco DCL4 and DCL2, respectively. While the 21-nt class of viral siRNAs was predominant in non-treated plants, 21-nt and 22-nt classes accumulated at almost equal (but low) levels in dsRNA treated plants, suggesting that dsRNA treatment may boost DCL2 activity. Taken together, our findings confirm the efficacy of topical application of dsRNA for plant protection against viruses and shed more light on the mechanism of antiviral RNAi.

## 1. Introduction

RNA interference (RNAi) is an evolutionarily conserved mechanism, present in eukaryotic organisms, that regulates gene expression via mRNA degradation, repression of translation, and chromatin remodeling [1]. It is involved in developmental regulation, stress response, or defense against invading nucleic acids like transposons or viruses [2]. Its role as a natural antiviral defense system in plants, invertebrates, and mammals has been well documented [3,4,5,6,7] and is considered together with the RNA decay and RNA quality-control pathways as ancestral forms of an antiviral immunity which may operate cooperatively for their antiviral function [8].

As our understanding of the RNAi mechanism deepens, the potential to exploit this mechanism in plant pest control has long attracted research interest, especially against viruses since preventive or control plant protection measures against viral diseases are very limited and the public pressure for adoption of environmentally-friendly crop protection strategies is constantly increasing. The main research focus in this direction has been the development of transgenic plants aiming at induction of RNAi that led to attenuation or elimination of viral disease symptoms. To this respect, diverse plant–virus pathosystems have been studied and crops engineered for virus resistance have been developed, using among others so-called RNAi transgenes designed to express dsRNA as a key trigger of RNAi [9,10]. Furthermore, certain engineered crops such as squash resistant to *Watermelon mosaic virus* 2, *Zucchini yellow mosaic virus* (ZYMV) and *Cucumber mosaic virus* (CMV), papaya resistant to *Papaya ringspot virus*, potato resistant to *Potato virus Y*, bean resistant to *Bean golden mosaic virus*, tomato, and sweet pepper resistant to CMV have been approved for commercial production [11].

However, as public opinion for transgenic plants in several parts of the world is not favorable and often even opposing, RNAi-induced resistance via alternative non-transgenic approaches is gaining ground. The topical application of RNA molecules on plants, also designated as plant ‘RNA vaccination’, is one of these approaches for induction of the RNAi mechanism in plants against viruses. Tenllado and Díaz-Ruíz [12] applied successfully by mechanical inoculation in vitro produced dsRNA molecules derived from viral sequences to ‘vaccinate’ *Nicotiana benthamiana* plants against *Pepper mild mottle virus* (PMMoV), *Tobacco etch virus* and *Alfalfa mosaic virus*, showing that these molecules can interfere with virus infection in a sequence-specific manner. Similarly, in vivo produced dsRNA molecules in bacterial cells promoted specific interference with the infection in *N. benthamiana* plants by PMMoV and *Plum pox virus* [13]. Many more plant–virus combinations have since been investigated regarding the effectiveness of topical application of dsRNA molecules against the cognate virus pathogen, as reviewed recently by Mitter et al. [14], Voloudakis et al. [15] and Dalakouras et al. [16].

In plant RNAi defense against RNA viruses, dsRNA intermediates of viral replication are processed by Dicer-like (DCL) family proteins DCL4 and DCL2 into 21- and 22- nucleotide (nt) small interfering RNAs (siRNAs), respectively. These siRNAs bind to Argonaute (AGO) family proteins and guide the resulting RNA-induced silencing complexes (RISCs) to target viral RNAs for cleavage and degradation or translational repression. DNA viruses are additionally targeted by nuclear DCL3 processing viral dsRNA generated by sense and antisense transcription into 24-nt siRNAs (reviewed in [17]). Transgenic expression of dsRNA cognate to both RNA and DNA viruses can confer virus resistance, likely by pre-inducing and/or boosting the antiviral RNAi responses (reviewed in [10]).

To counteract the plant antiviral RNAi-based mechanism and successfully induce disease, the plant viruses have evolved to encode proteins that act as suppressors of RNAi (viral suppressors of RNA silencing, VSRs). It has been shown that VSRs can interfere and block almost any stage of RNAi pathway [2]. Thus, a key issue determining the outcome of the race between a pathogenic virus and its host plant is when the RNAi is triggered by dsRNA.

In the present study, the dsRNA vaccination approach was applied against CMV, the type species of the genus *Cucumovirus* in the family *Bromoviridae*. CMV has a worldwide distribution and above all the broadest host range of any known plant virus, infecting more than 1000 species of plants, including monocots and dicots, herbaceous plants, shrubs, trees, agricultural crops, ornamentals and wild species [18]. CMV can be transmitted by at least 86 aphid species in a non-persistent manner, corresponding to the noncirculative mode [19,20]. Most of the infected hosts develop systemic mosaic symptoms, more or less severe depending on the host genotype and the strain, as well as mosaics of light and dark green areas on the infected leaves [21,22]. CMV disease outbreaks in tomato, pepper, or cucurbits, crops with high commercial interest, usually result in great economic losses [23,24,25,26]. The disease symptoms in tomato include leaf and plant shrinkage, upward leaf curling, vein purpling, chlorosis, pericarp hardening and discoloration, plant and fruit necrosis, and even plant death [20,23,24,25,27]. The common symptoms of CMV on tobacco plants (*Nicotiana tabacum* cv. ‘Xanthi’) are stunting, deformations, and mild mosaic on leaves, while a few CMV strains induce yellow mosaic on tobacco leaves [28,29]. So far, control of CMV depends mainly on the use of pesticides against insect vectors, resistant or tolerant varieties and cross-protection [23]. In addition, pathogen-derived resistance (PDR) has been demonstrated against CMV using various segments of the CMV genome [30].

The genome of CMV consists of three single-stranded positive-sense RNA species (RNA 1, RNA 2, RNA 3) and a subgenomic RNA (RNA 4) which is transcribed from RNA3 to serve as mRNA for the viral coat protein (CP). Viral CP encoding genes are the most frequently used genes in plant transformation for PDR and proved to be efficient in many cases [19,31,32]. CP-mediated resistance is achieved through post-transcriptional gene silencing (PTGS) and has been used to create CMV resistant plant species including tobacco, cucumber, tomato, melon, squash and pepper [31,33,34,35,36]. In one study, tobacco was transformed with an RNAi construct containing an inverted-repeat of a 747-bp fragment of the CMV CP separated by a spacer, and the obtained plant lines with two or more copies of the transgenes were resistant to CMV infection [37]. Likewise, *N. benthamiana* transgenic plants were produced with 100% resistance to CMV, using an RNAi transgene with CMV CP inverted-repeat sequences separated by an intron [38]. 

Apart from the viral CP genes, other gene targets used to engineer plant resistance include VSRs, such as the CMV 2b protein [39]. The CMV 2b protein is a multifunctional protein involved in host-specific, long-distance movement, symptom induction, and acts as a virulence determinant by suppressing plant RNAi and gene silencing pathways. CMV 2b inhibits siRNA-directed local and systemic silencing by sequestering siRNA duplexes and/or blocking antiviral activity of AGO1 (reviewed in [2]). Qu et al. [40] demonstrated that transgenic expression in tobacco of the 2b-specific artificial miRNA is an effective method to protect hosts from infection by CMV. Additionally, there have been reports of tobacco and tomato plants transgenic for CMV replicase showing a variable degree (0 to 100%) of resistance depending on the challenging CMV strain (subgroup) [31].

In the present study, we show that topical application of dsRNA derived from CP and 2b gene sequences of CMV can protect tobacco plants from systemic infection of CMV as well as reduce local lesions on leaves of the local host *Chenopodium quinoa*. Using deep small RNA sequencing and bioinformatics, we demonstrate that dsRNA-mediated interference with systemic CMV infection coincides with strongly reduced accumulation of 21- and 22-nt viral siRNAs and alteration in their ratio, indicative of changes in relative activities of DCL4 vs. DCL2.

## 2. Results

DsRNA molecules were produced using the in vitro and in vivo methods, previously described by Voloudakis et al. [41] and schematically depicted in Supplementary Figure S1. Both in vitro and in vivo dsRNAs for CMV CP (657 bps) and 2b (336 bps), resistant to DNase and RNase A treatments, were produced in sufficient quantities [3–4 µg/µL in vitro dsRNA-CP (dsCP) or in vitro dsRNA-2b (ds2b) or in vivo ds2b; 0.1–0.3 µg/µL in vivo dsCP; as estimated on agarose gel after RNase A/DNase I treatment, Supplementary Figure S2], and then tested for their protective effects against CMV infection in *Nicotiana tabacum* and *C. quinoa* plants.

### 2.1. dsRNA Treatments of Tobacco Plants Resulted in Variable Levels of Protection against CMV Infection, Which Depended on the dsRNA Construct Used and the Timing of Virus Inoculation

All *N. tabacum* plants treated with CMV inoculum alone (*n* = 157) or CMV inoculum in mixture with in vivo produced dsRNA-MalE (dsMalE) (*n* = 19), used as a control for sequence specificity of dsRNA, became infected after 14 days post inoculation (dpi), showing typical CMV symptoms including leaf curl/bump-like symptoms, mosaic symptoms, dwarfing of plants and necrosis of leaves. These results confirmed that the CMV inoculum used was infectious and the environmental conditions applied were appropriate for symptom development on tobacco plants. The dsMalE, that does not share sequence homology with CMV, did not prevent CMV infection, thus confirming the sequence specific nature of the antiviral RNAi. All 38 tobacco plants, mock-inoculated with sterile distilled water instead of CMV, remained healthy.

**Figure 1:**
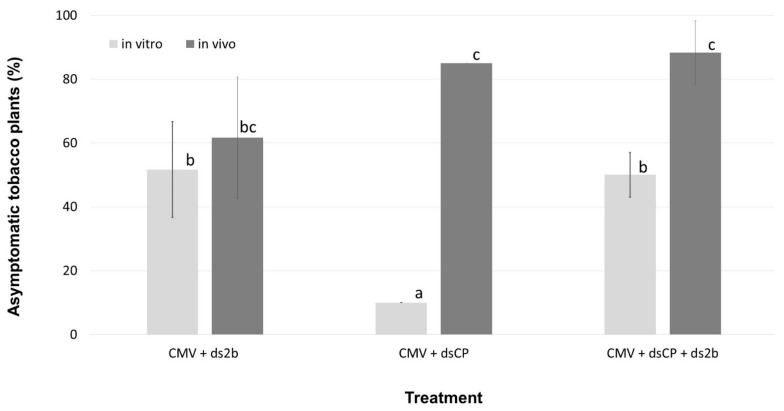
dsRNAs derived from CMV sequences confer a varied level of resistance to tobacco plants against the cognate virus. The histogram shows the percentage of asymptomatic plants determined for each treatment of tobacco plants with in vitro or in vivo produced dsRNA molecules. Data represent mean of the replicates ± standard deviation. Letters indicate the significant differences (*p* < 0.05) between the bars (Tuckey HSD test).

**Figure 2:**
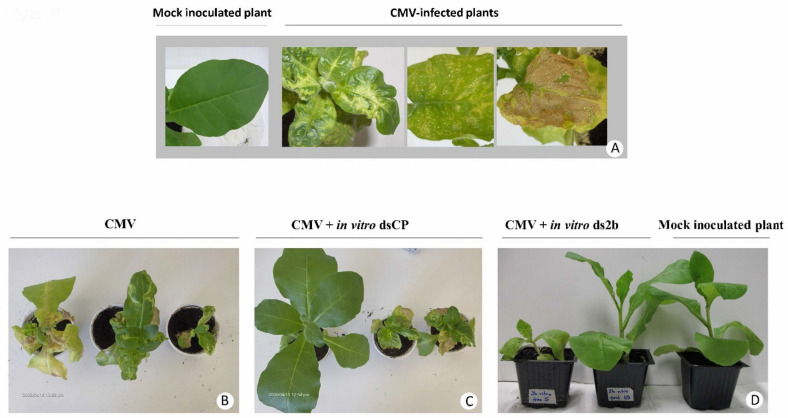
In vitro produced dsRNA molecules confer resistance to tobacco against CMV. (**A**) Range of symptoms of CMV-infected plants in comparison with the mock (water) inoculated plants. (**B**–**D**) Response of tobacco plants to the application of CMV jointly with in vitro produced dsRNA derived from the cognate virus. The tobacco plants were inoculated with: CMV (**B**), CMV jointly with in vitro dsCP (**C**) or CMV jointly with in vitro ds2b (**D**). Plants were photographed at 14 dpi.

**Figure 3:**
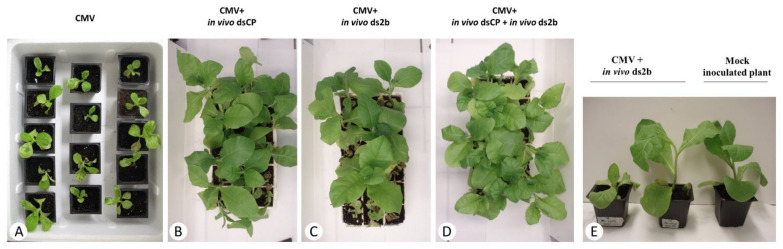
In vivo produced dsRNA molecules confer resistance to tobacco against CMV. Response of tobacco plants to the application of CMV jointly with in vivo produced dsRNA derived from the cognate virus. The tobacco plants were inoculated with: (**A**) CMV, (**B**) CMV jointly with in vivo dsCP, (**C**) CMV jointly with in vivo ds2b, (**D**) CMV jointly with in vivo dsCP and in vivo ds2b, (**E**) CMV jointly with in vivo ds2b in comparison with a mock (water) inoculated plant. Plants were photographed at 21 dpi.

When the dsRNA molecules had sequence homology with CMV, variable levels of protection against CMV infection were observed at 14 dpi in three repeated experiments. The co-application of in vitro produced dsCP together with CMV on a total of 60 plants provided 10% protection, while in vitro produced ds2b protected from CMV infection 35 to 65% of the co-inoculated plants (*n* = 60). When the in vitro produced dsCP and ds2b molecules were applied jointly, the protection level ranged from 45 to 55% of the co-inoculated plants (*n* = 40). For this treatment, the number of plants taken into account was 40 instead of 60 because in one replicate the infectivity of CMV in control plants was 75% rather than 100%, and thus this replicate was not further considered. In the case of in vivo produced dsRNA molecules, the application of dsCP protected from CMV infection 85% of co-inoculated plants (*n* = 60), while ds2b protected 40 to 75% of co-inoculated plants (*n* = 60). When both in vivo dsRNA molecules were applied simultaneously, the protection ranged between 80 to 100% of the co-inoculated plants (*n* = 60). (Figure 1, Figure 2 and Figure 3). Tukey HSD test showed that the protective effect of the in vitro ds2b was not significantly different to that of the in vivo ds2b or the joint application of in vitro ds2b and in vitro dsCP. Similarly, the in vivo ds2b was not significantly different to in vivo dsCP or to their joint application. On the contrary, in vitro dsCP was significantly less effective (*p* < 0.05) than any other treatment, and especially in comparison with the in vivo dsCP.

Regarding the time-course of symptom development recorded in one of the three repeated experiments, we observed that when the *N. tabacum* plants were inoculated only with CMV, 18% of the plants (*n* = 11) remained asymptomatic at 7 dpi and 0% of the plants were asymptomatic at 14 dpi. In contrast, for plants (*n* = 13) co-inoculated with CMV and in vitro ds2b, the asymptomatic plants were 77% at 7 dpi, 62% at 14 dpi, and 38% at 19 dpi. Thus, the application of in vitro ds2b delayed symptom development.

To test the protective action of the dsRNA molecules when applied on *N. tabacum* plants prior or after to CMV inoculation, the in vivo produced dsCP was selected, because it was the most protective in the co-inoculation experiments and applied either four days before or four days after CMV inoculation, or simultaneously with CMV. At 15 dpi, protection was observed only when the dsRNA molecules were applied simultaneously (*n* = 20) or 4 days before CMV inoculation (*n* = 20), with a percentage of asymptomatic plants being 65% and 35%, respectively. On the contrary, all plants (*n* = 20) were symptomatic when the application of dsCP was delayed for 4 days after CMV inoculation.

To test sequence specificity of the dsRNA protective effects, the in vivo produced ds2b was applied in mixtures with inoculum of unrelated viruses including Tobacco mosaic virus (TMV) (*n* = 20), Potato virus Y (PVY) (*n* = 20), or Tobacco rattle virus (TRV) (*n* = 20). All the treated plants became infected with the corresponding viruses after 21 days, displaying the characteristic disease symptoms for TMV, PVY, or TRV. In contrast, co-application of the in vivo ds2b in mixture with CMV, 55% of the co-inoculated tobacco plants (*n* = 20) remained asymptomatic after 21 days. This confirms the sequence specificity of ds2b protective effect against CMV, i.e., the virus from which the dsRNA sequence derived, but not against the unrelated viruses lacking any 2b gene homology.

### 2.2. dsRNA Treatment Protects from Local Infection in Chenopodium Quinoa Plants

We then tested dsRNA effects on local lesions of CMV infection on *C. quinoa*, a local lesion host plant of CMV. When leaves of *C. quinoa* were treated with CMV alone or CMV in mixture with in vitro dsRNA molecules, the average number of local necrotic lesions per leaf formed at 6 dpi was: 45 for CMV-treated leaves (*n* = 10), 14.5 for leaves treated with CMV and dsCP (*n* = 10), 2.6 for leaves treated with CMV and ds2b (*n* = 10), and 16.3 for leaves treated with CMV, dsCP and ds2b (*n* = 10) (Figure S3). Tukey HSD test analysis showed that the leaves treated with the dsRNA molecules exhibited a significantly (*p* < 0.01) lower number of lesions in comparison with the leaves treated only with CMV. Based on the same test, there was no significant difference (*p* < 0.05) between the protective effect of ds2b, dsCP, or their joint application. However, it is worth noting that the application of ds2b resulted in the lowest number of lesions per leaf, but this protective effect seems to be lost when it is jointly applied with dsCP, possibly due to dilution effect on this local host (Figure 1).

### 2.3. dsRNA Treatment Leads to Reduced Accumulation of Viral siRNAs and Alteration in Their Size Profile in Systemic Leaves

Systemic leaves from symptomatic (CMV-infected), asymptomatic (treated with in vivo dsCP or ds2b) and control (non-inoculated/non-treated) tobacco plants at 21 dpi were used for total RNA extraction, followed by analysis of viral siRNA accumulation, genome distribution and size-class profiles using small RNA blot hybridization and Illumina sequencing.

**Figure 4:**
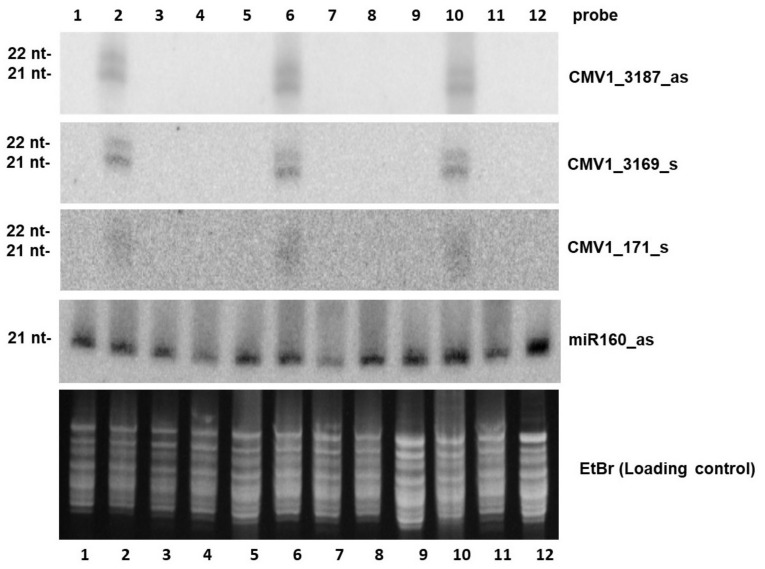
Small RNA blot hybridization analysis of CMV-infected, dsRNA treated and negative control tobacco plants. Total RNA samples resolved by electrophoresis on a 15% polyacrylamide gel stained with ethidium bromide (EtBr) and then transferred to a nylon membrane. The membrane was successively hybridized with p^32^-labeled DNA oligonucleotide probes specific to CMV RNA1-derived antisense (CMV1_3169_s and CMV1_171_s) and sense (CMV1_3187_as) siRNAs and plant miRNA160 (miR160_as). After each hybridization, the membrane was exposed to a phosphor screen for 20 hrs to 6 days and then scanned using a Phosphor Imager. The RNA samples are derived from: the 3rd leaf of mock inoculated plants (Lanes 1, 5, 9); a pool of leaves from plants inoculated with CMV showing symptoms (Lanes 2, 6, 10); the 3rd leaf (Lanes 3, 4, 7) or the 2nd leaf (Lane 11) or the 4th leaf (Lane 12) of a plant inoculated with CMV and treated with in vivo dsCP, not showing symptoms; the 3rd leaf of a plant inoculated with CMV and treated with in vivo ds2b, not showing symptoms (Lane 8).

Blot hybridization analysis using viral strand-specific oligonucleotide probes revealed accumulation of abundant, virus-derived 21–22 nt siRNAs of both polarities in all leaf samples from CMV-infected *N. tabacum* plants (Figure 4, lanes 2, 6, 10). In contrast, viral siRNAs were below detection level in systemic leaves of all the dsRNA-treated plants (Figure 4, lanes 3, 4, 7, 8, 11, 12) or the negative control plant (Figure 4, lane 1, 5, 9).

**Figure 5:**
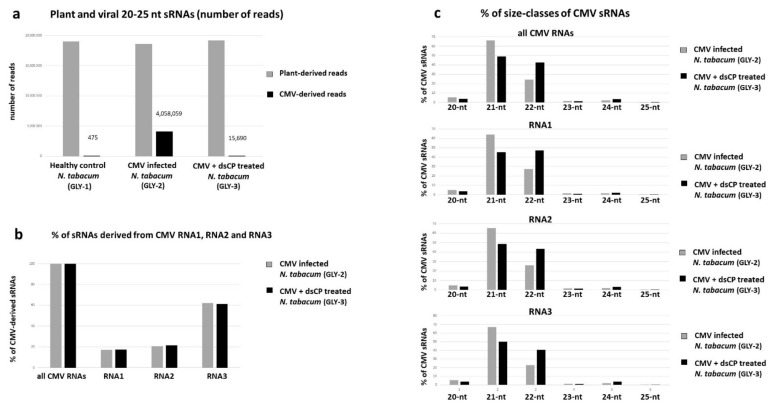
Illumina sequencing counts of endogenous and viral small RNAs (sRNAs) in healthy control (GLY-1), CMV-infected (GLY-2) and in vivo dsCP treated (GLY-3) tobacco plants. The 20- to 25-nt sRNA reads from GLY-1, GLY-2, and GLY-3 plants were mapped to the complete virus genome (CMV) or each of the individual CMV genomic RNA (RNA1, RNA2, RNA3) reference sequences with up to 2 mismatches and were counted. (**a**) The counts of CMV genome-derived 20–25 nt sRNA reads and total non-CMV reads in each library. (**b**) Percentage of each of the individual viral RNA-derived 20–25 nt sRNAs (RNA1, RNA2, RNA3) in the pool of the complete CMV genome-derived 20- to 25-nt sRNAs (CMV all). (**c**) Percentage of each size class in the 20- to 25-nt pools of the complete virus genome (CMV all)- and each genomic RNA (RNA1, RNA2, RNA3)-derived sRNA reads.

**Figure 6:**
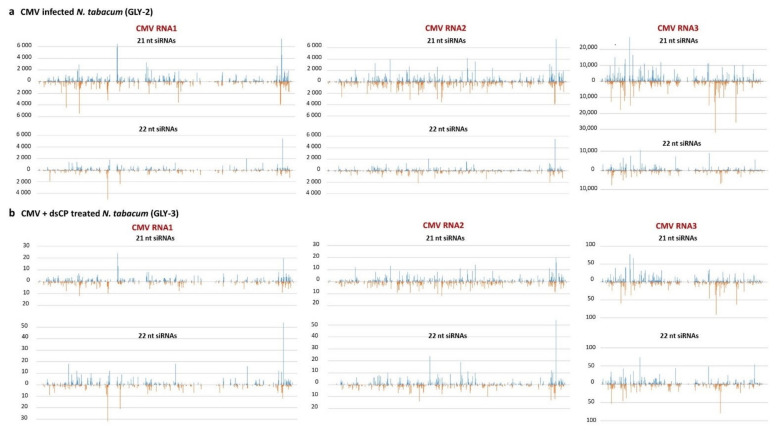
Single-nucleotide resolution maps of virus-derived small interfering RNAs (siRNAs) from: (**a**) CMV-infected (GLY-2) and (**b**) in vivo dsCP treated (GLY-3) tobacco plants. For each plant sample, the histograms plot the numbers of 21 and 22 nt viral siRNA reads at each nucleotide position of the CMV genomic RNAs RNA1 (3361 nt), RNA2 (3060 nt), and RNA3 (2216 nt) (mapped with up to 2 mismatches). The bars above the axis represent sense (forward) reads starting at each position and those below represent antisense (reverse) reads ending at the respective position.

Illumina small RNA sequencing analysis of selected samples of the CMV-infected, dsCP- treated and control plants revealed that viral siRNAs do accumulate in systemic leaves of the plant co-inoculated with CMV and dsCP, albeit at much lower levels (0.08% of total plant + virus reads) than in systemic leaves of the plant inoculated with CMV alone (17.90% of total reads) (Figure 5a). Only negligible number of viral reads were detected in the control plant (475 reads, or 0.002% of total reads which can be considered as the cross-contamination level between these three libraries sequenced in one lane of Illumina Genome Analyzer). Despite the drastic difference in accumulation levels, in both CMV-infected and dsRNA-treated plants viral siRNAs were derived from all three CMV genomic RNAs, with RNA3 being the biggest siRNA producer, followed by RNA2 and RNA1 (Figure 5b) and represented both sense and antisense strands with similar hotspot profiles between the plants (Figure 6a vs. Figure 6b). Notably, the size-class profile of viral siRNAs differed between the CMV-infected plant accumulating predominantly 21-nt size-class, followed by 3-times less abundant 22-nt class, and the dsRNA-treated plant accumulating 21-nt and 22-nt classes at almost equal levels (Figure 5c). Other size classes were underrepresented in both cases, consistent with the major role of DCL4 and DCL2 in RNAi-based defense against cytoplasmic RNA viruses such as CMV and previous studies on the biogenesis of CMV-derived siRNAs in Arabidopsis [42,43]. Despite the substantial alteration in the ratio of the two major size classes of CMV siRNAs (Figure 5c), the hot spot distribution along sense and antisense strands of the virus genome were similar for each size class between the plants (Figure 6a vs. Figure 6b), suggesting that dsRNA treatment may have altered relative activities of DCL4 and DCL2 rather than relative accumulation of viral siRNA precursors from the three genomic RNAs.

## 3. Discussion

From the first report by Tenllado and Diaz-Ruiz [12] until today, the topical application of RNA molecules has been tested against a series of plant viruses and viroids including *Alfalfa mosaic virus*, *Cymbidium mosaic virus*, *Pepper mild mottle virus*, *Plum pox virus*, *Papayaringspot virus*, *Pea seed borne mosaic virus*, *Potato virus Y*, *Sugarcane mosaic virus*, *Tobacco etch virus*, *Tobacco mosaic virus*, *Chryanthemum chlorotic mottle viroid*, *Citrus exocortis viroid*, *Potato spindle tuber viroid* (reviewed in [14,15]), *Zucchini yellow mosaic virus* [44], *Tomato leaf curl virus* [45] and more recently *Tomato spotted wilt virus* [46], and *Tomato yellow leaf curl virus* [47].

Regarding CMV, initial data on tobacco RNA vaccination employing in vitro and in vivo dsRNA were initially reported by Holeva et al. [48,49] and on pepper RNA vaccination employing in vivo ds2b by Borah et al. [50]. Likewise, Mitter et al. [51] reported that spray application of in vitro ds2b on leaves of cowpea plants conferred plant resistance to CMV, but only if the leaves were virally challenged within 5 days post-dsRNA application. However, when the ds2b was loaded on layered double hydroxide nanosheets as carriers, the sprayed tobacco plants remained protected for at least 20 days after CMV inoculation. Such a non-toxic, biodegradable and biocompatible clay-based matrix as carrier of dsRNA was used to enhance the stability of the dsRNA molecules on the leaf surface of the field crop, as well as to achieve gradual release of these molecules, thus managing longer protection of the treated plants against CMV infection. Although transgenic tobacco plants expressing the CMV CP were found to exhibit CMV resistance [37], there is no experimental evidence on a similar plant response in case of topical application of dsRNA homologous to CMV CP.

In this study, we investigated the efficiency of long dsRNA molecules since it has been suggested that short dsRNA molecules (e.g., having a length of 300 bp or less) exhibit reduced RNAi efficiency against plant viruses [12]. DsRNAs homologous to CMV CP (encoded by RNA 3) were compared to dsRNAs for CMV 2b (encoded by RNA 2) in their ability to protect tobacco plants against CMV. Both dsRNA molecules were produced by in vitro and in vivo methods, as previously described [41]. In the bioassays performed, it was observed that dsRNA targeted to CP and 2b of CMV conferred varied levels of protection against CMV, depending on the host plant and the dsRNA production method used (in vitro or in vivo). The protective impact of applying a combination of these two dsRNA molecules was also evaluated.

On tobacco, the application of in vitro dsCP (657 bp) conferred a much lower level of protection (ca. 10%) in comparison to 35–65% protection observed with the application of in vitro ds2b (336 bp); the combination of both in vitro dsRNAs did not result in higher levels of protection likely because the in vitro dsCP molecules exhibited very low protective effects. The two selected target genes are encoded by different genomic RNAs of CMV, which accumulate at different levels with the RNA3 and RNA4 carrying the CP sequence being much more abundant than the RNA2 carrying the 2b sequence. The threshold levels of the inducer dsRNAs may depend on the target RNA levels in the host cell cytoplasm that are key for the exhibition of PTGS. Degradation of low abundance target RNAs triggered by dsRNA treatment may have a greater effect in the final outcome of RNAi. Furthermore, the function of the protein encoded by the targeted RNA could also affect the protective effect. In order to clearly identify the reason for this difference one has to study the number of efficient siRNA molecules produced by the two dsRNAs [52].

Interestingly, the level of protection obtained by the application of in vivo dsCP was much higher (ca. 85%) than that of the in vitro dsCP (ca. 10%), and was even comparable to that of in vivo ds2b (40–75%). Furthermore, the application of a mixture of the two in vivo produced dsRNA molecules showed the highest protection efficiency (80–100%), a percentage that is extremely attractive. The difference in protection efficiency between in vitro and in vivo produced dsRNA molecules was mainly observed for those derived from the CP gene, which were designed over a longer sequence (657 bp) than the dsRNA molecules derived from the 2b gene (336 bp). It is possible that the in vivo produced dsRNA molecules may contain not only the expected full-length dsRNA molecules, but also shorter than expected dsRNA molecules due to aberrant transcription or degradation by nucleases in the bacterial cells. Indeed, bacterial expression of dsRNAs resulted in the production of several shorter dsRNAs in addition to the main expected product of 657 bp (Supplementary Figure S2). These shorter molecules may confer a higher level of protection and should be further investigated. In RNAi transgenic plants, both short and long dsRNA constructs were found to be protective; notably accumulation of transgene-derived 24-nt siRNAs coincided with higher levels of resistance and even total immunity to viral infection (reviewed in [10]). In the case of exogenous dsRNA application, it is possible that the cellular uptake of the different length molecules may also be a crucial factor for dsRNA’s bioactivity. Furthermore, Tabein et al. [46] suggested that the choice of the viral region targeted by dsRNAs is crucial to induce resistance, on the basis of their bioassay results showing that from two dsRNAs of similar size targeting the *N* and *NSm* genes of *Tomato spotted wilt virus*, respectively, only that silencing the *N* gene had a protective impact on *N. benthamiana* and tomato plants.

Overall, in our study, the application of the dsRNA molecules simultaneously with CMV on tobacco plants conferred a delay in symptom development. A bioassay involving application of in vitro ds2b showed that the percentage of asymptomatic plants at 7 dpi was 77% or 18%, with or without ds2b application, respectively, and at 14 dpi at 62% or 0%, respectively. The attempt to apply the in vivo dsCP molecules, which had shown the highest protection efficiency, four days before CMV inoculation, as a preventive action, showed that the protective effect was maintained, although at a lower level (35%) in comparison to the 65% observed when the dsRNA molecules were applied simultaneously to CMV inoculum. In addition, no therapeutic action was observed when the dsRNA molecules were applied four days after CMV inoculation. The maintenance of the protective effect of the applied dsRNA molecules has been documented in studies with other plants viruses. DsRNA targeting HC-Pro and CP genes of ZYMV conferred protection at least for 20 dpi [44], while dsRNA targeting the RP gene of PMMoV conferred resistance up to 70 dpi [13]. Several other studies on foliar application of dsRNAs to induce host resistance against a series of plant viruses have been performed, as reviewed recently by Dubrovina et al. [53].

Our study also confirmed sequence specificity of the dsRNA molecules since the topical application of in vivo ds2b on tobacco plants, which were challenged with unrelated viruses lacking a 2b homologue, i.e., TMV, TRV, and PVY, did not confer any resistance. In addition, the 808 bp dsMalE non-homologous to CMV did not confer any resistance in tobacco plants against CMV. Specificity of the dsRNA molecules is a crucial parameter when considering the induced RNAi as a prospective tool to control plant viral diseases. It is important within the crop plant itself but also towards exposed non-target organisms, e.g., beneficial insects and mammals. As research data accumulate proving successful control cases of pests and pathogens employing RNAi in agriculture, there is also a growing need to assess possible associated off-target risks [54,55,56,57,58,59,60,61].

Using *C. quinoa* plants in a local lesion assay, we found a significant decrease in the number of developed necrotic spots when the dsRNA molecules were applied together with CMV, suggesting that dsRNA treatment interferes with local spread of viral infection. Very early effects of dsRNA molecules to resistance were documented in tobacco-TMV interaction, with in vitro dsRNA for p126 gene counteracting the proteomic changes of tobacco induced by TMV infection as early as 15 min post infection [62]. Interestingly, in the present study, only low amounts of virus-derived siRNAs were detected in systemic leaves of tobacco plants exhibiting phenotypic resistance to CMV at 21 dpi, which may suggest that, upon treatment with dsRNA, the virus had not replicated efficiently in the treated cells to be able to efficiently move from cell to cell and systemically within the plant. Our deep sequencing analysis of viral siRNAs in systemic leaves of CMV-infected vs. dsRNA treated plants revealed that dsRNA treatment not only dramatically reduces the number of virus-derived siRNAs but also alters their size class profile in that 22-nt and 21-nt siRNAs become equally represented. This suggests that dsRNA treatment may boost production of 22-nt viral siRNAs. In Arabidopsis plants infected with CMV or other RNA viruses, DCL4, generating the 21-nt siRNAs, plays a primary role in antiviral defense, while DCL2, generating the 22-nt siRNAs, is a secondary dicer that takes over the antiviral defense when DCL4 is absent or inactivated [17,42,43]. It remains to be investigated if exogenous dsRNA treatment could induce DCL2 activity locally in the treated leaf tissues and systemically in non-treated leaves.

In the present study, we obtained data supporting the protection efficacy of the ‘RNA vaccination’ approach against CMV, one of the most destructive plant viruses, by applying dsRNA molecules directly onto crop and model plants. More research work is needed to optimize the delivery of the dsRNA molecules to host-plants, e.g., by conjugating them on matrices functioning as dsRNA carriers through the plant cell walls. Such novel matrices have already been developed including cell-penetrating peptides [63], clay nanosheets [14,51], cationic fluorescence nanoparticles [64] and DNA nanostructures [65]. In addition, testing the efficiency of dsRNA molecules under field conditions (e.g., [66]), where plants would be naturally infected, would provide strongest evidence on the feasibility of the approach that the small-scale bioassays performed in the present study have shown. It is important to note that RNA vaccination method was found applicable to aphid-transmitted virus [67]. Last but not least, addressing biosafety issues raised by such a biotechnological approach would reduce current uncertainties and contribute to the acceptance of this novel disease and pest control strategy. 

## 4. Materials and Methods

### 4.1. Virus Isolate, Bacterial Strains, and Plant Material

CMV-G2, a Hellenic isolate of CMV, obtained from a tomato plant (*Solanum lycopersicum* L.) exhibiting extreme leaf malformation and mottle, which was collected during a serious CMV disease outbreak in the area of Gastouni-Olympia (Greece) in 1998 [20], was used in this study. The isolate was preserved as dried tobacco leaf samples in CaCl_2_ and propagated in tobacco (*Nicotiana tabacum*) cv. Xanthi when needed.

The bacterial strain used for the in vivo production of dsRNA molecules was the *E. coli* strain HT115 (DE3), which has the RNase III gene disrupted by a Tn*10* transposon carrying a tetracycline-resistance marker, as well as an IPTG-inducible T7 RNA polymerase gene contained within a stable insertion of a modified lambda prophage λ DE3 [68]. For all other molecular biology manipulations, the *E. coli* strains Mach1 (Invitrogen, Carlsbad, CA, USA) or DH5α were used.

The bioassays were performed on *N tabacum* cv. ‘Xanthi’ and *Chenopodium quinoa* plants. Seeds were sown and young plantlets at the stage of two leaves were transplanted in 8-cm-diameter pots. Plants were placed in a growth chamber with constant environmental conditions (temperature/photoperiod: 24 °C for 14 h of light and 18 °C for 10 h dark).

### 4.2. Construction of the Target DNA Templates for Transcription

The primers and the corresponding PCR temperature profiles used in this study are shown in Table 1. To obtain the template DNA molecules for dsRNA production, total nucleic acids were extracted from tobacco artificially infected with CMV-G2, as described by Sclavounos et al. [20]. RT-PCRs were performed to obtain a cDNA fragment (*ca*. 850 bp) containing the CP gene with flanking regions and a cDNA fragment (*ca*. 500 bp) containing the 2b gene with flanking regions. In specific, first strand cDNA was obtained using 90 pmoles of primer (the 5′ CP primer for the cDNA fragment containing the CP gene or the CMV-2b-For2 primer for the cDNA fragment containing the 2b gene), 200 units of M-MuLV Reverse transcriptase (New England BioLabs, Ipswich, MA, USA) and 0.9 µg of total nucleic acids as template, in a 20 µL reaction. Then, 1 µL of the produced first strand cDNA was amplified in a 50 µL reaction using the proofreading Vent DNA Polymerase (New England BioLabs, Ipswich, MA, USA) and 0.2 µM of 5′CP/3′CP primers for the fragment containing the CP gene or CMV-2b-For2/CMV-2b-Rev primers for the fragment containing the 2b gene. The above two RT-PCR products were cloned into pCRIITopo (Invitrogen, Carlsbad, CA, USA) and transformed into *E. coli* Mach 1 Topo cells (Invitrogen, Carlsbad, CA, USA). The resulting plasmid constructs: pCRIITopo::CMV-CP_850_ and pCRIITopo::CMV-2b_500_ were confirmed by colony PCR and digestion with EcoRI.

For the in vivo transcription, two plasmid constructs were prepared: (a) one to produce dsRNA of a CP gene fragment (657 bp), and (b) one to produce dsRNA of 2b gene (336 bp): (a) The 657 bp fragment of CMV-G2 CP gene was amplified with primers CMV-CP-LIT-For and CMV-CP-LIT-Rev from plasmid pCRIITopo::CMV-CP_850_ (Supplementary Figure S1.i. step 1). The PCR product was gel excised, cloned into the pCRIITopo plasmid vector, and transformed into *E. coli* Mach 1 cells. The resulting plasmid construct pCRIITopo::CMV-CP_657_ was confirmed by EcoRI restriction digest, PCR with M13F/M13R primers and CMV-CP-LIT- For/CMV-CP-LIT-Rev primers (Supplementary Figure S1.i. steps 2, 3). The CP gene fragment was then excised by restriction digest with EcoRI and BamHI, gel purified, cloned in the plasmid cloning vector LITMUS28i (New England BioLabs, Ipswich, MA, USA) similarly digested with EcoRI and BamHI, and transformed into *E. coli* DH5a cells. The resulting plasmid construct pLITMUS28i::CMV-CP_657_ was confirmed by restriction digest with EcoRI and BamHI, colony PCR with CMV-CP-LIT-For/CMV-CP-LIT-Rev and T7 primers (Supplementary Figure S1.i. steps 4, 5, 6). (b) The CMV-G 2b gene (336 bp) was amplified using the primers CMV-2b-F- Linker and CMV-2b-R-Linker from plasmid pCRIITopo::CMV-2b_500_ (Supplementary Figure S1.ii. steps 1, 2). The PCR product was gel purified, and 1 µL of a 1:100 dilution of this purified PCR product was amplified with EcoRI-T7-Linker-BamHI primer (Supplementary Figure S1.ii. steps 3, 4). This PCR product was digested with NcoI and BamHI, ligated into plasmid cloning vector LITMUS28i (New England Biolabs, Ipswich, MA, USA) similarly digested with the same enzymes and transformed into *E. coli* DH5a cells. The plasmid construct pLITMUS28i::2b_336_ was confirmed by colony PCR with primers M13F/M13R and EcoRI-T7-Linker-BamHI primer (Supplementary Figure S1.ii. steps 5a, 6). The plasmid constructs pLITMUS28i::CMV-CP_657_ and pCRIITopo::CMV-2b_501_ were confirmed also by sequencing. The plasmid pLITMUS28iMal (New England Biolabs, Ipswich, MA, USA) was used as control plasmid. This plasmid carries the 808 bp BglII-EcoRI fragment of pMal-p2 vector (New England Biolabs, Ipswich, MA, USA) i.e., a non-functional portion of the maltose/maltodextrin-binding periplasmic protein encoding gene of *E. coli* (malE gene) with no sequence similarity with CMV. The three above plasmid constructs pLITMUS28i::CMV-CP_657_, pLITMUS28i::2b_336_, and pLITMUS28iMal were transformed into competent *E. coli* HT115(DE3) cells, prepared using the CaCl_2_ method [70], in order to be used for the in vivo production of dsRNA (Supplementary Figure S1.i. step 7 and Figure S1.ii. step 7).

For the in vitro transcription, two DNA templates were prepared in order to produce dsRNA of: (a) a 657 bp fragment of CP, and (b) the 2b gene (336 bp), all containing a T7 RNA polymerase promoter at the 5′ ends of both strands. Both DNA templates were prepared by a two-step PCR approach (Supplementary Figure S1.ii. steps 1 to 5b, Figure S1.iii.). The 1st PCR was performed with the primers corresponding to the targeted gene and the respective plasmid DNA template shown in Table 2. The PCR products were separated by 1.5% (*w*/*v*) agarose gel electrophoresis and gel purified. The 2nd PCR step was performed with primer EcoRI-T7- Linker-BamHI, and 1 µL of 1:100 gel purified PCR product from 1st PCR as DNA template. The PCR products were separated by 1.5% (*w*/*v*) agarose gel electrophoresis, gel purified, and used as DNA templates for in vitro transcription.

### 4.3. Production of dsRNA Molecules

In vitro and in vivo approaches were used for the production of dsRNA molecules for exogenous application in plants [41].

#### 4.3.1. In Vivo dsRNA Production

*E. coli* HT115(DE3) cells transformed with one of the plasmid constructs: pLITMUS28i::CMV-CP_657_, pLITMUS28i::2b_336_ or pLITMUS28i::Mal, were used for production of dsRNA molecules, as described previously by Tenllado et al. [13] with minor modifications [41]. Briefly, single colonies of *E. coli* HT115(DE3) transformants containing any one of the three above plasmid constructs were grown with shaking at 37 °C for 16 h in LB supplemented with tetracycline at 12.5 µg/mL and ampicillin at 100 µg/mL. Each culture was then diluted 1:100 to a final volume of 4.5 L of LB supplemented with the same antibiotics, allowed to grow at 37 °C to OD_595_ = 0.5, and then supplemented with IPTG to induce T7 polymerase for an additional 2 h. The final cell concentration obtained was OD_595_ = 1.25. Total nucleic acids were extracted from the bacterial cells of the above cultures by a phenol-chloroform extraction method and ethanol precipitation. The nucleic acids pellet was resuspended in 2.7 mL of DEPC-treated water and used for the plant assays.

#### 4.3.2. In Vitro dsRNA Production

The in vitro transcription reactions were set up using the T7 RiboMAX^TM^ Express RNAi System (Promega, Madison, WI, USA) and appx. 0.7 µg of one of the two PCR-generated DNA templates described above, derived from the CP (657 bp) and 2b (336 bp) genes, following manufacturer’s instructions. Incubation of the reactions at 37 °C was allowed for 3 h. The reactions were subsequently placed at 85 °C and left to slowly cool down to room temperature overnight. For confirmation by gel analysis, 1 µL of the transcription reactions was electrophoresed on 1.5% agarose gel.

Both the in vitro and in vivo produced RNAs were treated with DNase and RNase provided with the T7 RiboMAX^TM^ Express RNAi System (Promega) in 2xSSC buffer at 37 °C for 1 h to confirm quality and quantity of produced dsRNA of the targeted sequences. The treated dsRNA was submitted to standard phenol-chloroform-isoamyl alcohol purification, ethanol precipitation, and resuspension in the same volume of DEPC-treated water. The purified dsRNA was analyzed by electrophoresis on 1.5% agarose gel. The results are summarized in Supplementary Figure S2.

### 4.4. Plant Bioassays

#### 4.4.1. Bioassay on Tobacco

The bioassays were performed on tobacco (*N. tabacum* cv. ‘Xanthi’) plantlets at the stage of two true leaves in order to evaluate the protective effect against CMV infection of the dsRNA molecules produced in vitro or in vivo in this study. The CMV inoculum used in the bioassays was obtained from CMV-G2-infected *N. tabacum* plants [20], by grinding 0.5 g of young CMV-infected leaf tissue at 15 days post inoculation (dpi) in 2 mL DEPC-treated sterile distilled water, followed by a dilution of 1/50 *v*/*v* for application onto the plants. The plants were treated with CMV inoculum or CMV inoculum in mixture with one or both of the dsRNA molecules (ds2b, dsCP) produced in vitro or in vivo in this study. Application of these mixtures onto plants was performed by rubbing a carborundum-dusted leaf surface with 10 µL of the respective mixture (8 µL of 1/50 *v*/*v* dilution of CMV inoculum with 2 µL of dsRNA preparations (12–16 µg in vitro dsCP or in vitro ds2b or in vivo ds2b; 0.4–1.2 µg in vivo dsCP) per leaf. Symptom development was recorded at 7 and 14 dpi. Each topical application treatment was applied onto 20 plants, with two leaves per plant treated. Three independent replicates of the bioassay were performed per treatment. The negative controls used included: (a) sterile distilled water instead of CMV inoculum, and (b) in vivo produced dsMalE in mixture with CMV inoculum. In all bioassays, plants treated only with CMV were used as control to assess infectivity of the viral inoculum used. In all bioassays, plants treated only with CMV were used as control to assess infectivity of the viral inoculum used. Inoculated plants were maintained for 21 days in a growth chamber under insect proof conditions at 22 °C day/night temperatures and 16 h daylight. Plants were monitored for symptom development at 7 and 14 dpi.

A bioassay was also performed on *N. tabacum* plants to monitor the time needed for symptom development upon CMV infection in the presence or not of dsRNA molecules. In specific, 11 plants were treated with CMV inoculum and 13 plants with CMV inoculum in mixture with in vitro produced ds2b. Symptom development was recorded at 7, 14, and 19 dpi.

The protective action of the dsRNA molecules when applied on *N. tabacum* plants with a four-day difference to CMV inoculation was also investigated. To this end, the in vivo produced dsCP was applied simultaneously or four days before, or four days after CMV inoculation, and symptom development was monitored for 15 dpi. Each treatment was applied to 20 plants, with two leaves per plant treated.

The specificity of the in vivo produced ds2b was investigated using a non-cognate viral inoculum, namely TMV, PVY, and TRV prepared similarly to CMV. Specifically, tobacco plants were treated with: (a) CMV inoculum (20 plants), and (b) in vivo produced ds2b in mixture with TMV, PVY, or TRV inoculum (20 plants per dsRNA-virus combination).

The percentage of CMV-infected plants in the bioassays was determined at 14 dpi, unless otherwise mentioned, by symptom evaluation and ELISA testing (*Cucumber mosaic virus* DTL complete kit, LOEWE^®^, DE) (data not shown).

#### 4.4.2. Bioassay on Chenopodium Quinoa Plants

The CMV inoculum used in the bioassay was obtained from CMV-G2-infected *N. tabacum* plants as described above, and its 1/10 *v*/*v* dilution in DEPC-treated sterile distilled water was used for artificial inoculation of *C. quinoa* plants, a local lesion host of CMV. Ten leaves were used in total per treatment (five leaves per plant). Each leaf received 10 µL CMV inoculum or CMV inoculum in mixture with in vitro dsCP or in vitro ds2b or their combination, as described above for bioassays on tobacco. The plants were kept at 21 °C and the local leaf necrotic lesions due to viral infection were counted at 6 dpi.

#### 4.4.3. Small RNA Blot Hybridization Analysis

The method applied in this study for detection of viral siRNAs in *N. tabacum* plants treated with CMV or CMV in mixture with in vivo dsRNA molecules, had been previously developed at Institute of Botany in University of Basel (Switzerland) for other plant–virus pathosystems [71,72,73]. Total RNA was extracted from: (a) the 3rd leaf of mock inoculated tobacco plants (healthy plants); (b) a pool of leaves from plants inoculated with CMV exhibiting symptoms; (c) the 2nd, 3rd, and 4th leaf of a plant co-inoculated with CMV and in vivo dsCP, not showing symptoms; (d) the 3rd leaf of a plant co-inoculated with CMV and in vivo ds2b, not showing symptoms. All leaves were collected at 21 dpi, frozen in liquid nitrogen and stored at −80 °C. For each of the above cases, the total RNA extraction was performed twice, using each time 1 g of leaf tissue and the mirVana^TM^ miRNA isolation kit (Ambion, Austin, TX, USA), following the manufacturer’s protocol. All extracts were analyzed separately. The total RNA extracts were quantified by a UV-Vis spectrophotometer (NanoDrop, Thermo Scientific, Waltham, MA, USA). The samples were analyzed on a 15% polyacrylamide gel (19:1 ratio of acrylamide to bis-acrylamide, 8 M urea). The gel was run at 350 V for about 4 hrs and then stained with ethidium bromide to evaluate the integrity and the amount of the loaded RNA (Figure 5). The RNA was then transferred from the gel to a Hybond N^+^ nylon membrane by overnight electroblotting. Blot hybridization was performed overnight at 35 °C using one of the four DNA probes listed in Table 3 and standard procedures. The probes were end-labeled with ^32^P using the Polynucleotide Kinase (Roche, Penzberg, Germany) and purified through spin columns containing Sephadex™ G-25 DNA grade F resin (MicroSpin^TM^ G25 columns, GE Healthcare, Chicago, IL, USA), following the manufacturer’s recommendations. Depending on the applied probe, the signal was detected after 1 to 6 days exposure of a storage phosphor screen (GE Healthcare, Chicago, IL, USA) to the membrane, which was then scanned using a radioisotopic imaging system (Molecular Imager, BioRad, Hercules, CA, USA). 

The probes were designed based on Illumina sequencing data from the exact same RNA samples, acquired through collaboration with Fasteris SA (www.fasteris.com, Geneva, CH (accessed on 19 March 2021)) (see below). Two probes (Cmv1_3169_s and Cmv1_3187_as; 24 nt) are specific for ‘hot spots’ (a region that serves as a preferential source of siRNA production) in sense and antisense polarity on RNA1 and the other one (Cmv1_171_s; 25 nt) for the ‘cold’ spot on the same RNA1. As a loading control, a 21 nt probe was employed that hybridizes to and detects the evolutionarily conserved plant miRNA miR160.

#### 4.4.4. Illumina Sequencing and Bioinformatics Analysis of Viral siRNAs

Total RNA samples from systemic leaves of the representative mock-inoculated healthy (GLY-1), CMV-infected (GLY-2) and in vivo dsCP treated (GLY-3) *N. tabacum* plants described above were selected for library preparation and Illumina small RNA sequencing using TruSeq^™^ SBS v5 kit and Genome Analyzer HiSeq 2000, respectively, at Fasteris SA. All the three libraries were bar-coded and sequenced in one lane of the HiSeq2000, yielding 19′002′747 (GLY-1), 22′658′565 (GLY-2), and 19′195′087 (GLY-3) reads. Following adapter trimming and low quality read removal, 20–25 nucleotide reads were mapped (MAQ) to the reference sequences of CMV RNA1 (NCBI Genbank accession CMU20220), RNA2 (CMU20218), and RNA3 (CMU20219), with up to 2 mismatches and counted based on size (20, 21, 22, 23, 24, 25, total 20–25 nt) and polarity (forward, reverse, total) using in-house Fasteris scripts to create single-nucleotide resolution maps of viral siRNAs. The resulting maps were transferred to Excel and further processed to create graphs and histograms shown in Figure 6.

## 5. Conclusions

This study presents the application of RNA vaccination in *N. tabacum* and *C. quinoa* against CMV, a virus with the largest host range. DsRNA molecules for the CP and 2b genes of CMV protected tobacco upon their topical application onto leaf surfaces. DsCP exhibited a four-day protective effect but no therapeutic effect was observed. The in vivo-produced dsCP was more efficient than the in vitro-produced dsCP which unexpectedly showed the smallest protective effect. In vitro ds2b was found to be more efficacious than in vitro dsCP. Deep small RNA sequencing analysis revealed that systemic leaves of dsCP treated plants accumulated low amounts of virus-derived siRNAs with an altered ratio of 21- and 22-nt size classes, suggesting that dsRNA treatment may boost the antiviral activity of DCL2.

## Figures and Tables

**Table 1:** Primers and PCR conditions used in this study.

Name of Primer	Sequence 5′ to 3′	PCR Thermal Profile	Reference
5′CP	CTCGAATTCGGATCCGCTTCTCCGCGAG	(94 °C for 3 min) × 1 cycle, (94 °C for 1 min, 50 °C for 1 min, 72 °C for 1 min) × 35 cycles, (72 °C for 10 min) × 1 cycle	[69]
3′CP	GGCGAATTCGAGCTCGCCGTAAGCTGGATGGAC		[69]
CMV-2b-For2	AGGGTTGAGCGTGTAAATTCC	As with 5′CP/3′CP oligonucleotides	This work
CMV-2b-Rev	CCGT(AT) AGCTGGATGGACAACC		This work
CMV-CP-LIT-For *	GCGGAATTCTCATGGACAAATC	(94 °C for 5 min) × 1 cycle, (94 °C for 1 min, 65 °C for 1 min, 72 °C for 1 min) × 35 cycles, (72 °C for 10 min) × 1 cycle	This work
CMV-CP-LIT-Rev *	GCGGGATCCGTTCAAACTGG		This work
T7	TAATACGACTCACTATAGG	(94 °C for 3 min) × 1 cycle, (94 °C for 30 sec, 50 °C for 30 sec, 72 °C for 30 sec) × 25 cycles, (72 °C for 5 min) × 1 cycle	New England Biolabs
CMV-CP-F-Linker **	GGGGATCCATGGACAAATCTGAATC	(98 °C for 30 sec) × 1 cycle, (98 °C for 10 sec, 60 °C for 30 sec, 72 °C for 30 sec) × 35 cycles, (72 °C for 10 min) × 1 cycle	This work
CMV-CP-R- Linker **	GGGGATCCTCAAACTGGGAGCAC		This work
CMV-2b-F-Linker **	GGGGATCCATGGAATCGAACGAAG	(98 °C for 30 sec) × 1 cycle, (98 °C for 10 sec, 62 °C for 30 sec, 72 °C for 1 min) × 35 cycles, (72 °C for 10 min) × 1 cycle	This work
CMV-2b-R-Linker **	GGGGATCCTCAAAACGCACCTTC		This work
EcoRI-T7-Linker- BamHI *^,^#	GAGAATTC*TAATACGACTCACTATAGG***GGATCC**	(98 °C for 30 sec) × 1 cycle, (98 °C for 10 sec, 55 °C for 30 sec, 72 °C for 30 sec × 35 cycles, (72 °C for 10 min) 1 cycle	This work
pUC/M13 Forward	CGCCAGGGTTTTCCCAGTCACGAC	(94 °C for 5 min) × 1 cycle, (94 °C for 1 min, 55 °C for 1 min, 72 °C for 1 min) × 35 cycles, (72 °C for 10 min) × 1 cycle	General primer
pUC/M13 Reverse	AGCGGATAACAATTTCACACAGGA		General primer

* The EcoRI restriction site (GAATTC) is underlined, and the BamHI restriction site (GGATCC) is bold and underlined. ** The Linker sequence is written in bold # The T7 promoter sequence is written in italics.

**Table 2:** Primer pairs and PCR conditions used to generate DNA templates for in vitro transcription.

DNA Template for In Vitro Transcription	Primer Pair Used in 1st Step PCR	DNA Template Used in the 1st PCR *
CP fragment of 657 bp	CMV-CP-F-Linker CMV-CP-R-Linker	pCRIITopo::CP_657_
2b (336 bp)	CMV-2b-F-Linker CMV-2b-R-Linker	pCRIITopo::2b_500_

* The lower script refers to the length of the cloned fragment, i.e., in pCRIITopo::CP_657_ means a 657 bp fragment of the CP gene; in pCRIITopo::2b_500_ means a fragment of 500 bp containing the 2b gene with flanking regions.

**Table 3:** DNA probes used for RNA blot hybridization.

Name of the Probe	Sequence (5′ to 3′)	Number of Nucleotides
Cmv1_3169_s *	TCCATCCAGCTTACGGCTAAAATG	24
Cmv1_3187_as *	GATTTCTCCACGACTGACCATTTT	24
Cmv1_171_s	GTTGATAAGACAGCTCATGAGCAGC	25
miR160a_as	TGGCATACAGGGAGCCAGGCA	21

* s = sense, as = antisense.

## Supplementary Materials

The following are available online at https://www.mdpi.com/article/10.3390/plants10050963/s1, Figure S1: Schematic representation of the methods for in vivo and in vitro production of dsRNA, Figure S2: Production of dsRNA molecules to be used for topical application in the bioassays., Figure S3: In vitro dsRNA-CP and in vitro dsRNA-2b provide protection against CMV in a local lesion assay.
