# Supplementary material for: Topical Application of Double-Stranded RNA Targeting 2b and CP Genes of Cucumber mosaic virus Protects Plants against Local and Systemic Viral Infection
Source: Plants (Basel). 2021 May 12;10(5):963. doi: 10.3390/plants10050963 (PMC8151262; doi:10.3390/plants10050963)
Supplement: Supplementary file 1 [file plants-10-00963-s001.zip › plants-1121816-supplementary.pdf]

Supplementary Material

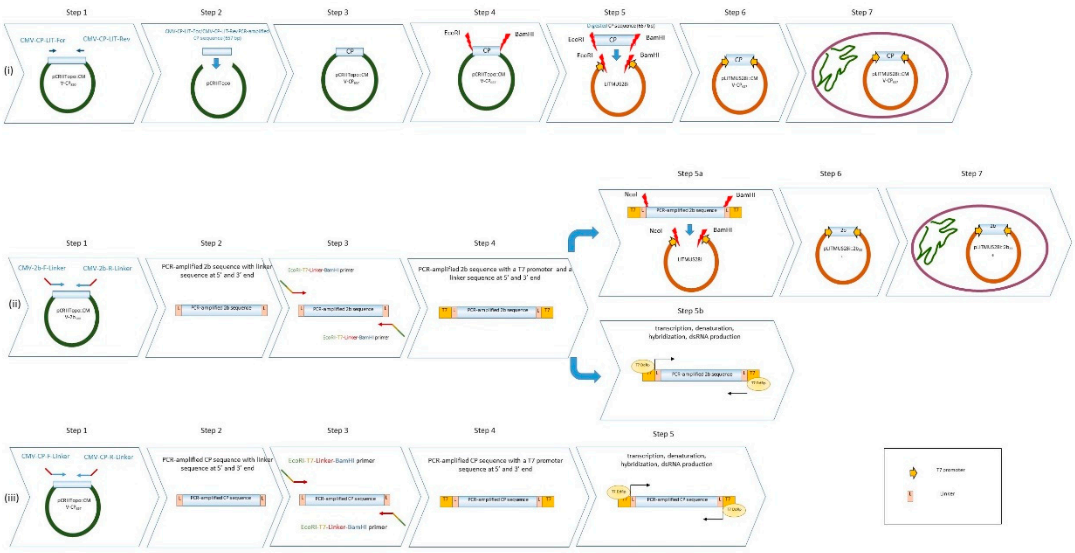

Figure 1. Schematic representation of the methods for in vivo and in vitro production of dsRNA. For each method, the main steps to generate the template for transcription are indicated. (i) In vivo production of dsCP following steps 1 to 7; (ii) In vivo production of ds2b following steps 1 to 7 via step 5a, and in vitro production of ds2b following steps 1 to 5b; iii) In vitro production of dsCP following steps 1 to 5. Diagrams are not drawn to scale.

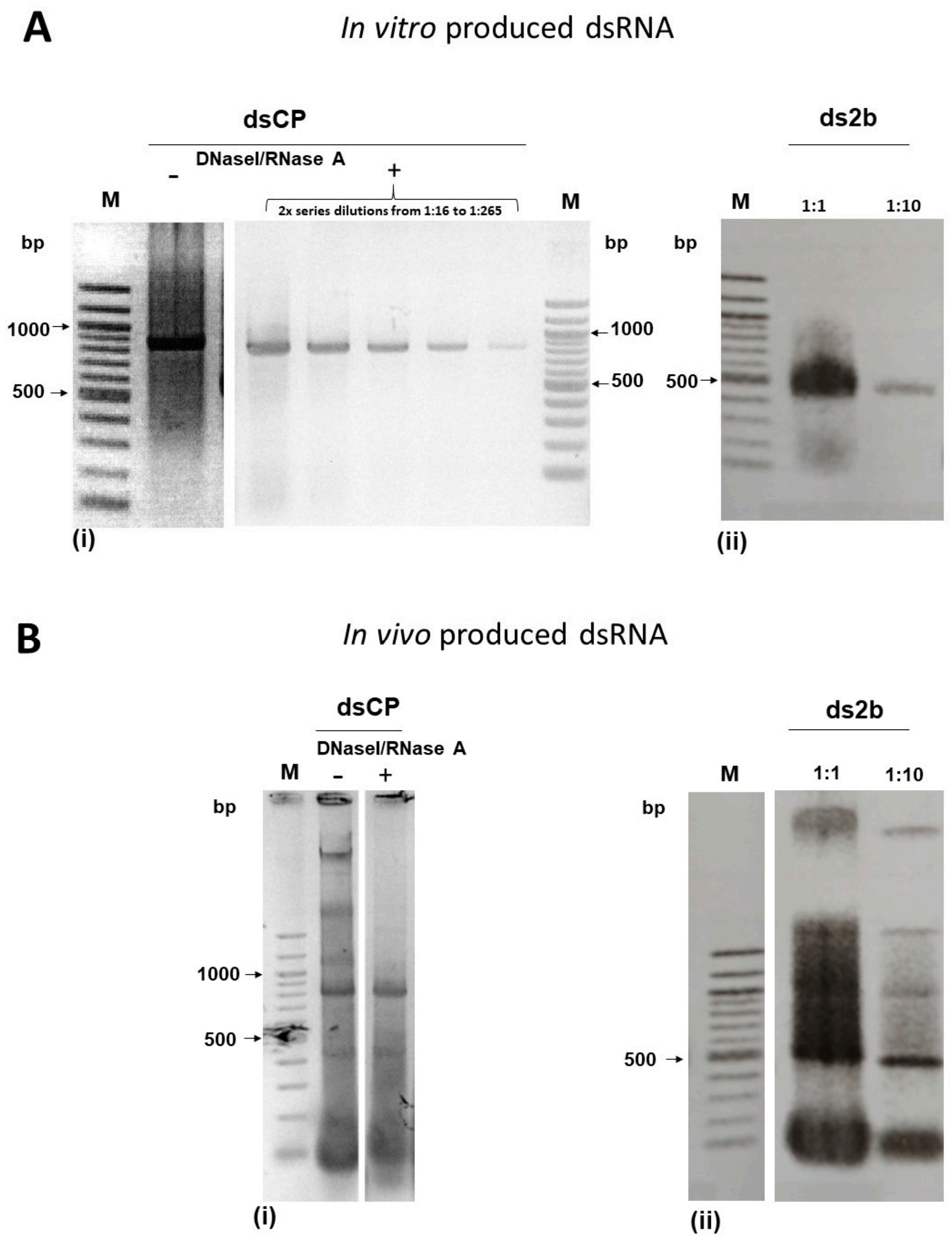

Figure S2. Production of dsRNA molecules to be used for topical application in the bioassays. A) In vitro production: (i) dsRNA derived from the CP gene of CMV (675 bp), as product of the transcription reaction with no further treatment (lane: '-') or after treatment with DNaseI/RNase A (lanes: '+') loaded as 2x series dilutions from 1:16 to 1:256). (ii) dsRNA derived from the 2b gene of CMV (336 bp), as product of the transcription reaction loaded as 1;1 and 1:10 dilutions. B) In vivo production: (i) dsRNA derived from the CP gene of CMV (675 bp), as crude extract from bacterial cells with no further treatment (lane '-') or after treatment with DNaseI/RNase A (lane: '+'). (ii) dsRNA derived from the 2b gene of CMV (336 bp), as crude extract from bacterial cells loaded as 1:1 and 1:10 dilutions. The DNA ladder (lane 'M') used is a 100-bp DNA ladder (New England Biolabs, USA).

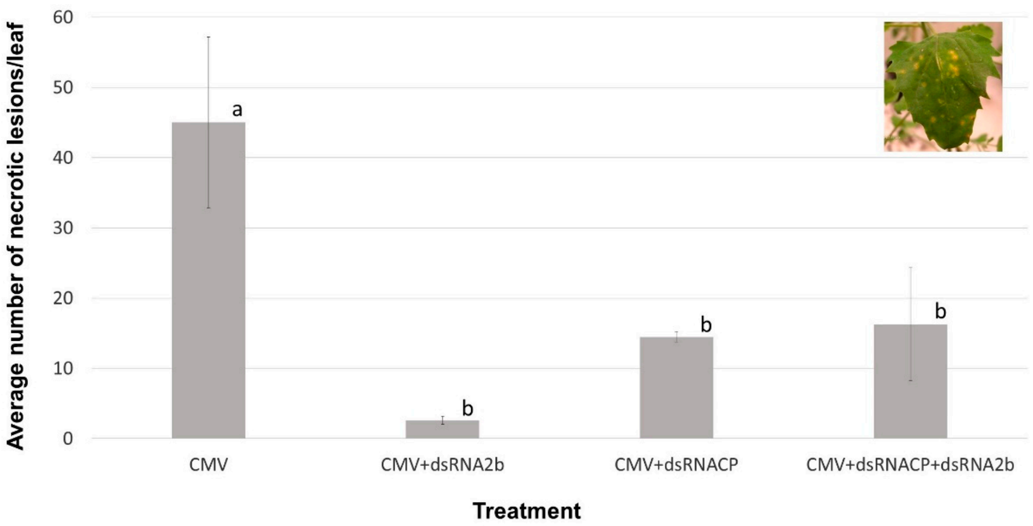

Figure S3. In vitro dsCP and in vitro ds2b provide protection against CMV in a local lesion assay. The histogram shows the average number of local necrotic lesions formed per leaf of *Chenopodium quinoa* (inset photo), upon inoculation with CMV, CMV jointly with in vitro dsCP, CMV jointly with in vitro ds2b or CMV jointly with in vitro dsCP and in vitro ds2b (n=10 leaves per treatment). The plants were kept at 21 °C and the local leaf necrotic lesions caused by CMV were counted at 6 dpi. Data represent mean ± standard deviation. Letters indicate the significant differences ($p<0.01$) between the bars (Tuckey HSD test).
